# Paclitaxel loading in cationic liposome vectors is enhanced by replacement of oleoyl with linoleoyl tails with distinct lipid shapes

**DOI:** 10.1038/s41598-021-86484-9

**Published:** 2021-03-31

**Authors:** Yuhong Zhen, Kai K. Ewert, William S. Fisher, Victoria M. Steffes, Youli Li, Cyrus R. Safinya

**Affiliations:** 1 College of Pharmacy, Dalian Medical University, Dalian, 116044 Republic of China; 2 Materials, Physics, and Molecular, Cellular, and Developmental Biology Department, University of California, Santa Barbara, CA 93106 USA; 3 Materials Research Laboratory, University of California, Santa Barbara, CA 93106 USA

**Keywords:** Drug delivery, Nanotechnology in cancer, Biophysics, Membrane structure and assembly

## Abstract

Lipid carriers of hydrophobic paclitaxel (PTX) are used in clinical trials for cancer chemotherapy. Improving their loading capacity requires enhanced PTX solubilization. We compared the time-dependence of PTX membrane solubility as a function of PTX content in cationic liposomes (CLs) with lipid tails containing one (oleoyl; DOPC/DOTAP) or two (linoleoyl; DLinPC/newly synthesized DLinTAP) *cis* double bonds by using microscopy to generate kinetic phase diagrams. The DLin lipids displayed significantly increased PTX membrane solubility over DO lipids. Remarkably, 8 mol% PTX in DLinTAP/DLinPC CLs remained soluble for approximately as long as 3 mol% PTX (the solubility limit, which has been the focus of most previous studies and clinical trials) in DOTAP/DOPC CLs. The increase in solubility is likely caused by enhanced molecular affinity between lipid tails and PTX, rather than by the transition in membrane structure from bilayers to inverse cylindrical micelles observed with small-angle X-ray scattering. Importantly, the efficacy of PTX-loaded CLs against prostate cancer cells (their IC50 of PTX cytotoxicity) was unaffected by changing the lipid tails, and toxicity of the CL carrier was negligible. Moreover, efficacy was approximately doubled against melanoma cells for PTX-loaded DLinTAP/DLinPC over DOTAP/DOPC CLs. Our findings demonstrate the potential of chemical modifications of the lipid tails to increase the PTX membrane loading while maintaining (and in some cases even increasing) the efficacy of CLs. The increased PTX solubility will aid the development of liposomal PTX carriers that require significantly less lipid to deliver a given amount of PTX, reducing side effects and costs.

## Introduction

**Figure 1:**
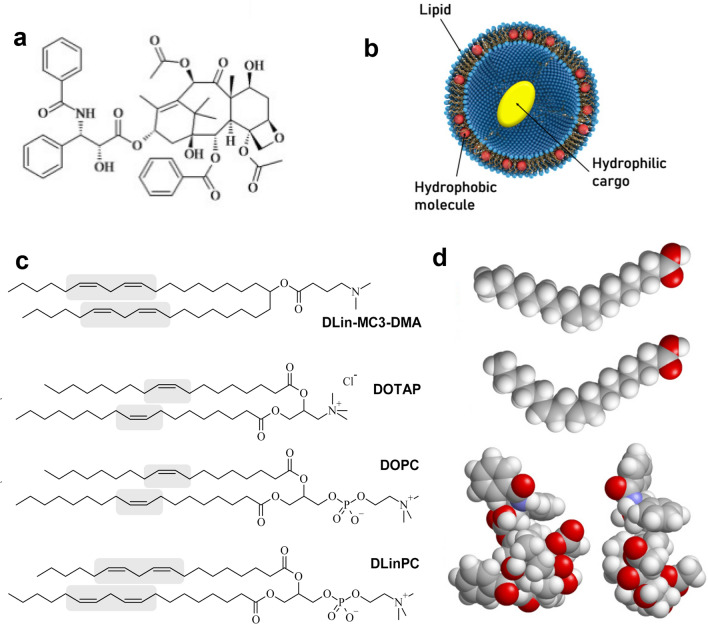
Paclitaxel (PTX) and lipid vectors. (**a**) Chemical structure of PTX. (**b**) A unilamellar liposome consisting of a self-assembly of amphiphilic lipid molecules. The liposome can carry hydrophobic molecules (red spheres) within its hydrophobic bilayer and hydrophilic molecules (yellow oval) in its aqueous interior. (**c**) Chemical Structures of DLin-MC3-DMA (the cationic lipid used in patisiran), DOTAP, DOPC (the lipids used in the Endo-TAG formulation of PTX) and DLinPC. The *cis* double bonds in the lipid tails are highlighted. (**d**) Space filling molecular models of the ground state structure of oleic acid (C18:1) and linoleic acid (C18:2) together with two views of the structure of PTX for size comparison. The structures were rendered using RasTop 2.2 (https://www.geneinfinity.org/rastop/).

Despite immense progress in treatment options and their effectiveness over recent decades, cancer remains a leading cause of death. Thus, there is an ongoing need for high-efficacy cancer chemotherapy with reduced side effects. Paclitaxel (PTX, Fig. 1a, c)^1^ is a potent and widely used (> $1 billion/year) cancer drug for treating ovarian, breast, lung, pancreatic, and other cancers^1–10^. PTX inhibits mitosis by stabilizing microtubules, which are inherently dynamical in vivo, and subsequently activates apoptotic signaling pathways that lead to cell death^1–3, 5, 11–14^.

Because PTX is hydrophobic and poorly soluble in water, it has to be delivered by a carrier (vector)^15, 16^. However, the carrier employed in the prevalent PTX formulation Taxol^17^, polyoxyethylated castor oil and ethanol, has been linked to severe hypersensitivity reactions requiring premedication^18–20^. Development of more efficient and safer PTX carriers has been an ongoing challenge for decades^21–27^. Albumin-bound PTX is an example of earlier success in carrier development and was approved by the FDA in 2005 (Abraxane; a nontargeted nanoparticle formulation). This formulation appears to have fewer adverse reactions than Taxol and eliminates drug-carrier toxicity, but reports on whether it improves patient survival are mixed^21, 28–30^.

Increasing the capacity (PTX loading) of the carrier is desirable because it means less carrier is required for a given PTX dose, reducing both cost and side effects stemming from the carrier. Furthermore, developing PTX carriers with higher efficacy, i.e., lower IC50 of PTX cytotoxicity, also reduces drug-related side effects because less PTX is required to exert its cytotoxic effect. Finally, based on its biochemical mechanism of action, PTX should be effective against most cancer cells. Therefore, development of novel, improved vectors for PTX which, for example, may be able to deliver PTX to an expanded range of tissues, could also open treatment avenues against an expanded range of cancers. For example, Abraxane appears to be effective to treat metastatic melanoma, whereas Taxol is not^31^.

Liposomes are highly versatile and widely studied carriers of hydrophilic as well as hydrophobic drugs in therapeutic applications, in particular for cancer^21, 32–46^. Most widely-known liposomal formulations, such as Doxil and Myocet, contain the cancer drug doxorubicin in the interior of the liposome (yellow oval in Fig. 1b). This is not feasible for PTX, however. PTX is much more hydrophobic (logP = 3.96) than doxorubicin (logP = 1.3), which can even be administered directly, without a solubilizing agent. In addition, the doxorubicin formulations rely on design principles that do not translate to PTX, because PTX lacks the functional groups that permit doxorubicin to be loaded via pH- or ion-gradient loading methods (forming reversibly soluble crystals within the liposomal aqueous pocket)^47^. Instead, hydrophobic drugs such as PTX are solubilized by and incorporated into the nonpolar (hydrocarbon chain) bilayer membrane of lipid-based carriers (red spheres in Fig. 1d)^16, 37, 48–50^.

Cationic liposomes (CLs; consisting of mixtures of cationic and neutral lipids) are particularly attractive as a lipid-based carrier for PTX because positively charged particles have been shown to passively target the tumor neovasculature^38, 40, 43, 51–56^, which has a greater negative charge than other tissues^39, 53^. Moreover, CLs are desirable as a lipid-based carrier for PTX because CLs are a prevalent nonviral vector (investigated as alternatives to engineered viruses) for the delivery of therapeutic nucleic acids (NAs, e.g., plasmid DNA or siRNA; electrostatically condensed with membranes with cationic headgroups)^34, 57–81^. This enables the use of CLs for combination therapies. The CL formulation EndoTAG (aka SB05), which served as a starting point for our investigations, has completed Phase II clinical trials and is currently in phase III trials^26, 52^. EndoTAG consists of CLs of the univalent cationic lipid DOTAP (2,3-dioleoyloxypropyltrimethylammonium chloride) and neutral DOPC (1,2-dioleoyl-*sn*-glycero-3-phosphatidylcholine) loaded with PTX (50:47:3 molar ratio)^37, 38, 40, 41, 82, 83^.

Because PTX is loaded in the bilayer of liposomes by hydrating a mixture of the lipid and PTX, the initial “loading efficiency” is 100%. However, if the amount of PTX in the membrane is larger than the membrane solubility limit, PTX precipitates out of the membrane and forms crystals over time. Once PTX has phase-separated into stable, water-insoluble crystals, the drug loses efficacy^37, 48, 84–86^. Thus, it is crucial that PTX remains soluble in the membrane on timescales relevant for delivery. Relatively few studies have investigated the PTX solubility limit in different types of membranes, and not many common themes have emerged^49, 50, 87–92^. Nearly all animal studies and clinical trials with liposome–PTX carriers have been conducted at 3 mol% PTX content^37, 38, 41, 82, 83, 93, 94^, the first reported membrane solubility limit of PTX^95^. Rarely, liposomal PTX formulations with higher membrane solubility have been reported, but they did not alter the structure of the lipid tails and did not provide a systematic approach to increase PTX loading^89, 96^.

The PTX membrane solubility strongly depends on lipid tail structure because the location of the drug within the bilayer implies that tails exhibiting favorable local packing interactions with PTX will suppress PTX self-association, nucleation, and crystal growth. PTX is quickly expelled from membranes consisting of chain-ordered saturated lipid tails or those that have a high concentration of cholesterol^37, 49, 50, 87, 88^. Lipids with chain-melted mono-unsaturated tails, on the other hand, are used in many of the lipid-based PTX carriers in development, such as EndoTAG, LEP-ETU, and DHP107^21, 93^. In this work, we instead focused on tails with multiple *cis* double bonds, because these increase chain disorder and modify chain flexibility. We hypothesized that this increased chain disorder and altered flexibility would affect molecular affinity to PTX compared to tails with one *cis* double bond. Currently there are only a few instances of commercial therapeutics containing lipids with poly-unsaturated fatty acid tails^32, 97^. A notable exception is DLin-MC3-DMA (Fig. 1c), the cationic lipid component of patisiran (Onpattro)^98^, which became the first FDA-approved siRNA therapeutic in 2018. We note, however, that the siRNA component interacts with the lipid’s headgroup in the case of patisiran and thus, unlike in our system, the active ingredient has no direct interactions with the polyunsaturated tails. (As an aside, poly-unsaturated fatty acids have been studied as therapeutic entities in and of themselves for their anti-oxidant properties^99, 100^).

We pursued the development of CL carriers with a tail structure that improves solubility of PTX in their hydrophobic membrane. Such vectors require less lipid to deliver a given amount of PTX, reducing costs and side effects. Carriers with high solubility have also shown increased efficacy^48^, allowing administration of lower total doses of PTX. We obtained promising initial results of increased PTX membrane solubility using CLs prepared from DOTAP (oleoyl (C18:1) tails; see Fig. 1c) and commercially available DLinPC (linoleoyl (C18:2) tails; see Fig. 1c) at a molar ratio of DOTAP/DLinPC/PTX = 30/70–x_PTX_/x_PTX_^101^. Encouraged by these results, we synthesized the univalent cationic lipid DLinTAP from linoleic acid and used it to prepare PTX-loaded CLs containing lipids with exclusively C18:2 tails.

We used differential-interference-contrast (DIC) microscopy to directly observe PTX crystal formation and generate kinetic phase diagrams, characterizing the time-dependence of PTX solubility as a function of PTX content for CLs with lipid tails containing either one (DOTAP/DOPC) or two (DLinTAP/DLinPC) *cis* double bonds. Using cell viability measurements, we then compared the efficacy of PTX-loaded DLinTAP/DLinPC and DOTAP/DOPC CLs in PC3 (prostate) and M21 (melanoma) human cancer cell lines and determined the IC50 for PTX cytotoxicity of these vectors in the same cell lines.

Replacing tails bearing one *cis* double bond (DO lipids) with those bearing two (DLin lipids) significantly increased PTX membrane solubility in CLs. Remarkably, 8 mol% PTX in DLinTAP/DLinPC CLs remained soluble for approximately as long as 3 mol% PTX in DOTAP/DOPC CLs. At the same time, the IC50 of PTX cytotoxicity against PC3 cells was unaffected by changing the lipid tails, and toxicity of the lipid carrier was negligible. In M21 cells, efficacy was not just maintained but approximately doubled for PTX-loaded DLinTAP/DLinPC CLs over DOTAP/DOPC CLs.

Small-angle X-ray scattering (SAXS) allowed determination of the self-assembled nanostructures of PTX-containing CLs, mixed with DNA as a condensing agent to improve the SAXS signal, at varying amounts of DLinPC and DOTAP. The structures begin to transition from lamellar (L_α_^C^) to inverse hexagonal (H_II_^C^) as the content of DLinPC is increased to 70 mol% DLinPC and beyond. However, kinetic phase diagram studies show that PTX membrane solubility decreases in membranes with dioleoyl tails upon transition from the lamellar to the H_II_ phase by replacing DOPC with DOPE in DOTAP-containing CLs. These results suggest that the significantly improved PTX membrane solubility in DLinTAP/DLinPC CLs is caused by enhanced affinity of PTX to linoleoyl compared to oleoyl tails, rather than by the structural transition from lipid bilayers to inverse cylindrical micelles.

Taken together, our findings show that CLs with suitable tail structure can increase the PTX membrane loading from the typically used 3 mol% to approximately 8 mol%, and that the efficacy of those CLs is as high or twice as high as the Endo-TAG formulation for PC3 cells and M21 cells, respectively. This will aid the development of liposomal PTX carriers that use less lipid, reducing side effects and costs.

## Materials and methods

### Materials

Stock solutions of DOPC, DOTAP, and DLinPC in chloroform were purchased from Avanti Polar Lipids. PTX was purchased from Acros Organics and dissolved in chloroform to 10.0 mM concentration. Calf thymus DNA was purchased from Thermo Scientific. DLinTAP was synthesized as described in the Supplementary Information.

### Cell culture

The human prostate cancer cell line PC3 (ATCC number: CRL-1435) and human melanoma cell line (M21) were a gift from the Ruoslahti Lab (Sanford Burnham Prebys Medical Discovery Institute). M21 cells are a subclone of the human melanoma line UCLA-SO-M21 derived in the lab of R. Reisfeld (Scripps Institute, La Jolla) and were originally provided by D. L. Morton (UCLA). Cells were cultured in DMEM (Invitrogen) supplemented with 10% fetal bovine serum (Gibco) and 1% penicillin/streptomycin (Invitrogen). Cells were cultured at 37 °C in a humidified atmosphere with 5% CO_2_ and split at a 1:5 ratio after reaching $\ge$ 80% confluency (every 48–72 h) during maintenance.

### Liposome preparation

Suspensions of sonicated and unsonicated liposomes at a total molar concentration (lipid + PTX) of 1 mM for cell viability experiments, 5 mM for DIC microscopy, and 30 mM for small-angle X-ray scattering measurements were prepared as described previously^48^.

In brief, stock solutions of individual lipids and PTX in organic solvent (chloroform/methanol; 3:1, v/v; same or higher concentration as that of the aqueous suspension to be prepared) were combined in small glass vials to yield the appropriate molar ratios (indicated in the text, figure, or figure legend; typically, the cationic lipid content remained constant within a series of samples, while the amount of neutral lipid was reduced as the amount of PTX was increased). After evaporation of the organic solvent by a stream of nitrogen for 10 min, the resulting lipid/PTX films were dried in a vacuum (rotary vane pump) for 16 h and hydrated with high-resistivity water (18.2 MΩ cm) to the desired concentration (see above). Immediately thereafter, suspensions were agitated with a tip sonicator (Sonics and Materials Inc. Vibra Cell, set to 30 W output) for 7 min to form small unilamellar vesicles (“sonicated liposomes”). We note that PTX loading with this liposome preparation method is quantitative (until PTX crystallizes as summarized by the kinetic phase diagrams), because PTX is a highly hydrophobic molecule and is thus incorporated within the membrane (red sphere rather than yellow ellipsoid in Fig. 1b).

### DIC microscopy (PTX solubility and kinetic phase diagrams)

Samples were prepared and assayed as described previously^48^, with the following modifications: the sample solutions were stored at room temperature for the duration of the experiment and imaged at 20 or 40 × magnification every 2 h until 24 h, every 12 h until 48 h, and daily thereafter until PTX crystals were observed. The kinetic phase diagrams report the time at which PTX crystals were observed in 2 of 3 independently prepared samples at each PTX content.

### Small-angle x-ray scattering

Samples for X-ray scattering were prepared by combining and vortexing 50 µL of a 30 mM aqueous liposome suspension with DNA solution (3.5 mg/mL in water) at a cationic lipid to DNA charge ratio of 1 in a 500 µL centrifuge tube. Following centrifugation in a table-top centrifuge at 5,000 rpm, the resulting pellets were transferred to quartz capillaries (Hilgenberg) with the help of excess supernatant. The capillaries were then centrifuged in a capillary rotor in a Universal 320R centrifuge (Hettich) at 10,000 g and 25 °C for 30 min. After centrifugation, the capillaries were sealed with a fast-curing epoxy glue.

SAXS measurements were carried out at the Stanford Synchrotron Radiation Laboratory, beamline 4–2, at 9 keV (λ = 1.3776 Å) with an Si(111) monochromator. Scattering data was measured by a 2D area detector (MarUSA) with a sample to detector distance of ≈3.5 m (calibrated with silver behenate). The X-ray beam size on the sample was 150 µm in the vertical and 200 µm in the horizontal directions. Scattering data is reported as azimuthally averaged scattering intensity in *q*-space.

### Cell viability assays

Cells were plated at a density of 5000 cells/well in 96-well plates and incubated overnight. Suspensions of sonicated liposomes were diluted in DMEM to reach the desired PTX concentration. The culture medium was removed from the wells by aspiration with a pipette, taking care not to aspirate any cells, and a total of 100 µL of the liposome suspension in DMEM was added to each well. Cells were incubated for 24 h before the liposome suspension was removed by manual pipetting and replaced with cell culture medium. After incubation for 48 h, the cell viability was measured using the CellTiter 96 AQueous-One Soution Cell Proliferation Assay (Promega). The assay solution was diluted sixfold with DMEM, and a total of 120 µL of this solution was added to each well. After 1 h of incubation, absorbance was measured at 490 nm using a plate reader (Tecan M220). Each data point shown comprises four identically treated wells and is normalized to the absorbance obtained for untreated cells.

Liposomes with different PTX contents were added to cells such that the resulting PTX concentration per well was identical for each data point, independent of the formulation. Because of this, the lipid concentration at each data point varies between formulations with different PTX contents^48^. To determine the IC50 values, we used the solver add-in for Microsoft Excel to perform a nonlinear least squares fit of the cell viability data to the equation y = A + B/(1 + [x/C]^D^). Here, y is the measured (normalized) cell viability, x is the total PTX concentration, A is the minimum cell viability, (A + B) is the maximum cell viability (B is the range of y), C is the IC50 (the concentration of PTX at which cell viability is halfway between the maximum and minimum values, i.e. where y = A + B/2), and D is the “slope factor” of the curve (indicating how steeply the viability declines). The minimum and range of cell viabilities (A and B) was given by the data, while C and D were used as fitting parameters.

## Results and discussion

### Lipid synthesis

**Figure 2:**
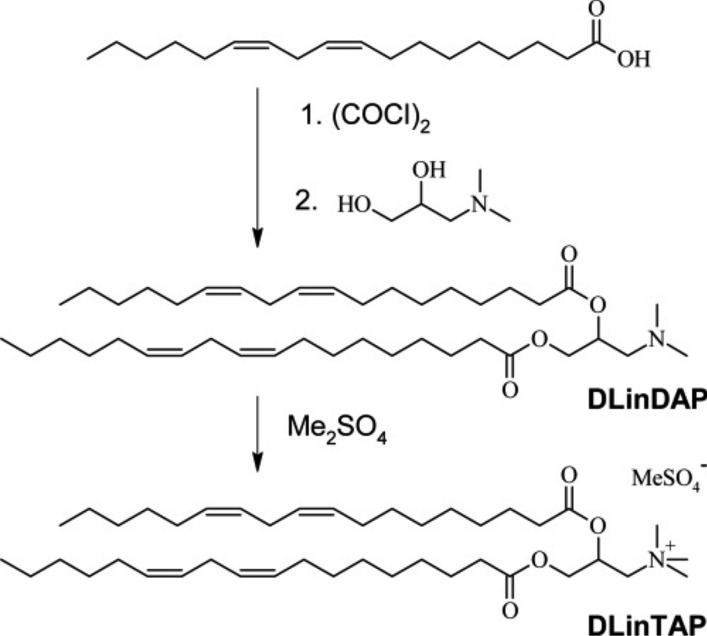
Synthesis of DLinTAP. The full details of the synthesis are reported in the Supplementary Information.

To be able to prepare CLs with tails that exclusively bear two *cis* double bonds (derived from linoleic acid, C18:2 $\Delta$ ^9^), we synthesized DLinTAP as shown in Fig. 2. The full details of the synthesis, which used a route analogous to that reported for DOTAP^102^, are reported in the Supplementary Information.

### PTX membrane solubility

Upon hydration of films prepared from a mixture of cationic lipid (DLinTAP or DOTAP), neutral lipid (DLinPC or DOPC) and PTX, PTX-loaded CLs formed spontaneously. These CLs were studied directly (unsonicated CLs; uni- and multi-lamellar with a broad distribution of larger sizes and an average diameter of ≈800 nm) or after sonication (small unilamellar CLs with diameter < 200 nm). As mentioned in the Introduction, the initial “loading efficiency” is 100%, but, depending on the composition, PTX may phase-separate and crystallize over time, decreasing the PTX loading of the CLs and reducing the amount of PTX that is effective against cancer cells.

#### DIC microscopy

**Figure 3:**
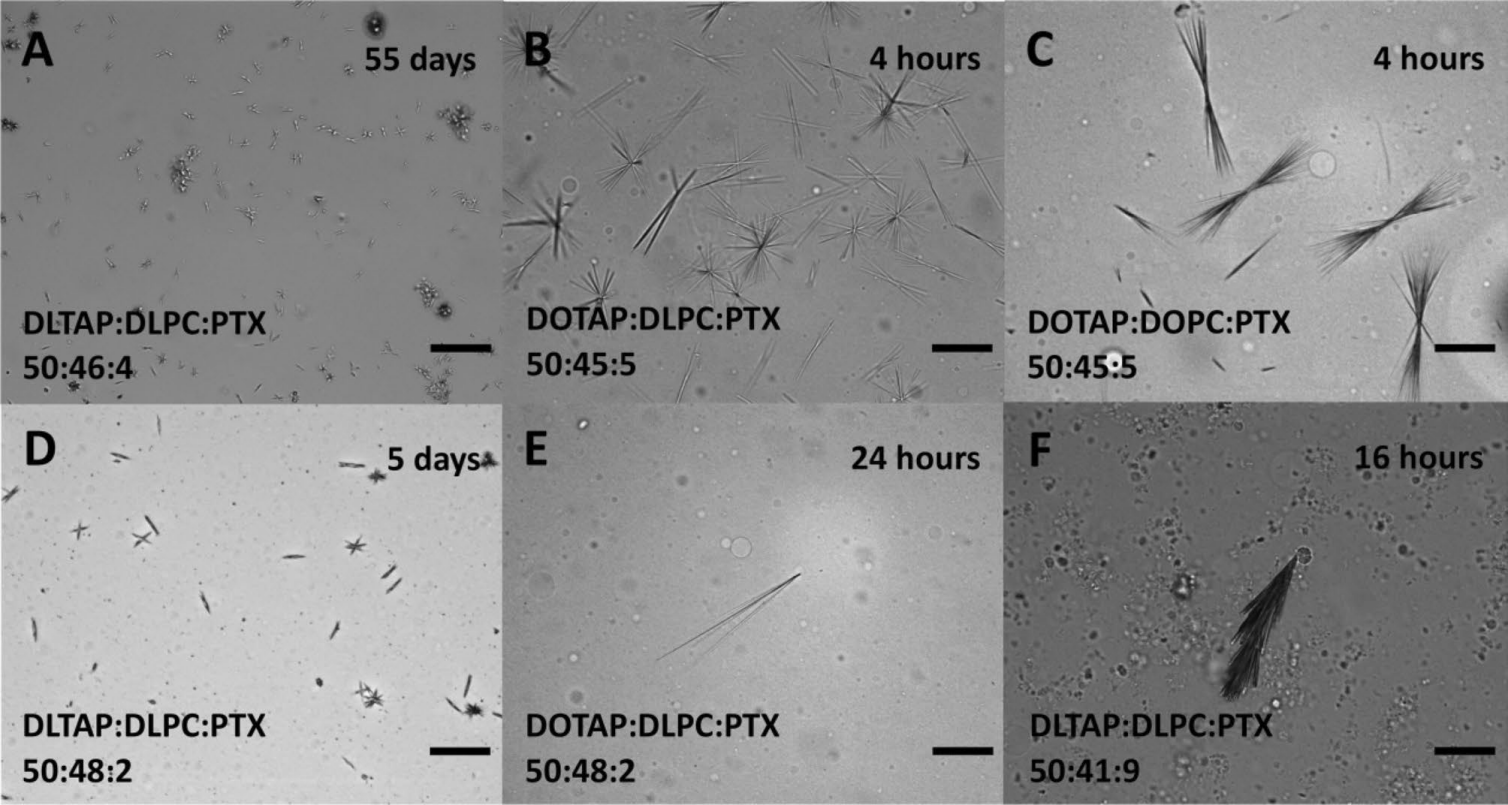
Selected DIC microscopy images of liposomes and phase-separated PTX, illustrating the size and shape variety of observed PTX crystals as discussed in the text. The samples were unsonicated PTX-loaded CLs of the molar compositions indicated on the images. Images were taken after hydration for the time noted. Scale bars: 50 μm.

To compare the solubility of PTX in CLs prepared from DLin- and DO-lipids, we used differential interference contrast (DIC) microscopy^48^. Starting 2 h after sample hydration, samples were observed at regular time intervals to check for PTX crystals as evidence of phase separation. Figure 3 shows DIC micrographs illustrating the variety of size and shape in the observed crystals (for unsonicated samples). In addition to characteristic needle-shaped PTX crystals (Fig. 3A, D, E), we commonly observed star and double-fan shapes (Fig. 3B, D and Fig. 3C, respectively). As PTX content increased, the number of crystals increased and their size decreased (compare Fig. 3B to Fig. 3E). Aggregates of PTX crystals were common at contents above 6 mol%, including the feather-like crystals observed at 9 mol% (Fig. 3F). The aspect ratio of PTX crystals from DLinTAP/DLinPC CLs was typically much smaller than that of crystals formed from DOTAP-containing CLs (compare Fig. 3A, D to Fig. 3B, C, E). The overall smaller size and larger number of PTX crystals from DLinTAP/DLinPC CLs suggests that there are fewer nucleation and growth sites in DOTAP-based samples.

#### Kinetic phase diagrams of PTX-loaded CLs

**Figure 4:**
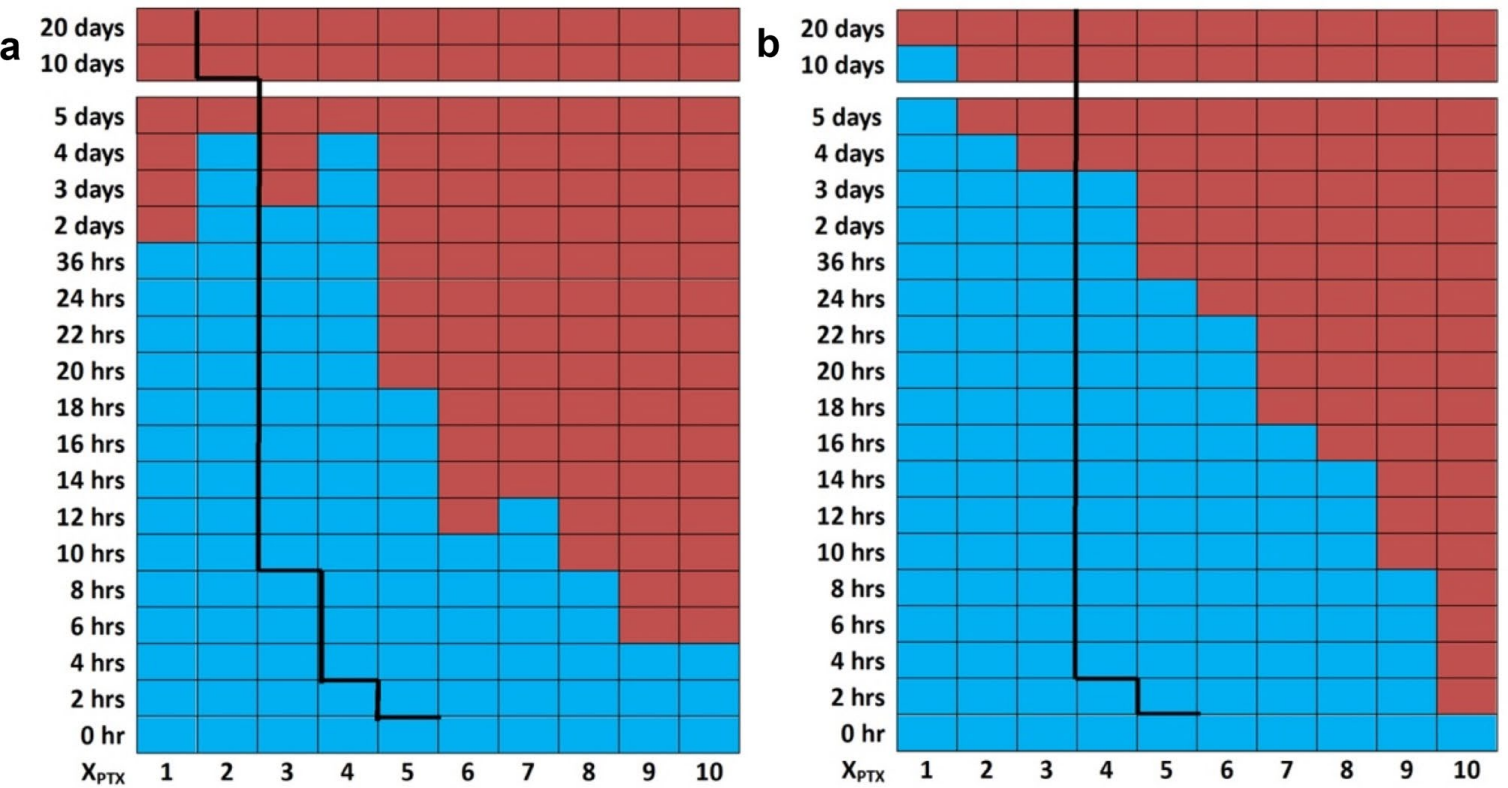
Kinetic phase diagrams of PTX solubility in PTX-loaded CLs with DLin (di-linoleoyl, C18:2) lipid tails (DLinTAP/DLinPC/PTX mole ratio = 50/50–x_PTX_/x_PTX_). DIC microscopy (Fig. 3) was used to detect PTX crystallization at the indicated times after hydration. The blue color indicates absence of PTX crystals (i.e., PTX remained soluble in the membranes), while the red color indicates presence of PTX crystals. The boundary separating the blue region from the red region is termed the PTX membrane solubility boundary. As a reference to facilitate comparison, the black lines show the solubility boundary for PTX-loaded CLs with monounsaturated DO (di-oleoyl, 18:1) tails (DOTAP/DOPC/PTX molar ratio = 50/50-x_PTX_/x_PTX_)^48^. The change in tail structure strongly affects PTX membrane solubility. (**a**) Kinetic phase diagram for sonicated PTX-loaded DLinTAP/DLinPC CLs. (**b**) Kinetic phase diagram for unsonicated PTX-loaded DLinTAP/DLinPC CLs.

The time-dependent PTX solubility data as a function of PTX content (kinetic phase diagrams) of sonicated and unsonicated PTX-loaded DLinTAP/DLinPC CLs (DLinTAP/DLinPC/PTX mole ratio = 50/50–x_PTX_/x_PTX_), as mapped out by DIC microscopy, are shown in Fig. 4a, b, respectively. Blue color indicates that PTX remained solubilized in the CL membranes, i.e., no crystals were observed. Red color indicates time points at which PTX crystals were observed. (Time points after the first observation of crystals were marked with red color even if no further samples were assessed, since crystallization from the membrane is irreversible.) For example, in the sonicated sample containing 10 mol% PTX (DLinTAP/DLinPC/PTX mole ratio = 50:40:10), crystals were first observed at the 6 h time point, meaning that PTX crystallized between 4 and 6 h after hydration (Fig. 4a). The line separating the blue and red regions of the kinetic phase diagram, which marks the onset of crystal formation, is termed the PTX membrane solubility boundary. For comparison, the black lines in Fig. 4a, b indicate the membrane solubility boundary for PTX-loaded DOTAP/DOPC CLs (where x_PTX_ = 3 is the Endo-TAG composition)^48^.

It is immediately obvious from inspection of the kinetic phase diagrams that the switch from DO- to DLin-tails dramatically improves the solubility of PTX in the CL membrane. For sonicated CLs (Fig. 4a), for example, a PTX content of 8 mol% remained solubilized in DLinTAP/DLinPC CLs for as long as a content of 3 mol% PTX did in DOTAP/DOPC CLs. The duration of PTX solubility in unsonicated CLs at most PTX contents is longer than in sonicated CLs, independent of the tail structure. However, CLs with DO tails show a solubility threshold at 3 mol% PTX content, meaning that PTX had crystallized from CLs with PTX content of 4 mol% at 4 h after hydration for both sonicated and unsonicated samples. (This is consistent with the reported PTX membrane solubility limit of 3 mol% used for most liposomal PTX vectors.) In contrast, the kinetic phase diagrams show a much more gradual decrease in the time for which PTX stayed solubilized with increasing PTX content for CLs with DLin tails. This is especially noticeable for unsonicated liposomes (Fig. 4b), where PTX remained soluble in DLinTAP/DLinPC CLs for over 3 days at 4 mol% and for over 22 h (but less than 24 h) at 6 mol%. Even at 9 mol%, PTX only crystallized between 8 and 10 h after hydration. What is impressive is that for sonicated CLs (similar in size and size distribution to preparations used in clinical trials) at 8 mol% PTX, crystals appeared on average only between 8 and 10 h after hydration. This relatively long time of PTX solubility should be sufficient for most in vivo applications.

Only at low loadings (PTX content < 2 mol% and < 3 mol% in sonicated and unsonicated samples, respectively) and long incubation times was PTX more soluble in DOTAP/DOPC CLs than in DLinTAP/DLinPC CLs. This may be due to oxidation of the DLin tails, because we took no special precautions to exclude oxygen from the small sample volumes during the repeated withdrawing of aliquots for DIC microscopy over time.

### Small-angle x-ray scattering

To investigate the effect of tail saturation on the structure of CL membranes, we used synchrotron small-angle X-ray scattering (SAXS) to determine the structure of CLs prepared from mixtures of DOTAP with either DOPC or DLinPC. To enhance the signal-to-noise ratio, we condensed the CLs with DNA. This has been shown to result in CL–DNA complexes where the equilibrium self-assembled structure of the membrane within the complex is determined by the spontaneous curvature (*C*_0_) of the lipid self-assembly^34, 103–105^. The spontaneous curvature is, in turn, determined by the average shape of the lipid molecules^106^.

DOPC/DOTAP CLs mixed with DNA form the lamellar (L_α_^C^) phase because both DOPC and DOTAP have a cylindrical shape with *C*_0_≈0 (see Fig. 6)^103^. We expected that DLinPC could be capable of forming the inverse hexagonal (H_II_) nonbilayer structure due to the increase in unsaturation from one to two *cis* double bonds in the lipid tails. The two *ci*s double bonds in DLinPC induce kinks in the lipid tails that can not readily be offset by *gauche* conformations in the single bonds, leading to the tails taking up a bigger lateral area compared to the headgroup area (Fig. 6). This results in an inverted-cone molecular shape as depicted in Fig. 6, corresponding to negative spontaneous curvature (*C*_0_ ˂ 0). A previous study on soy PC (a lipid mixture largely composed of DLinPC) using x-ray scattering and cryogenic TEM supports this hypothesis^107^.

**Figure 5:**
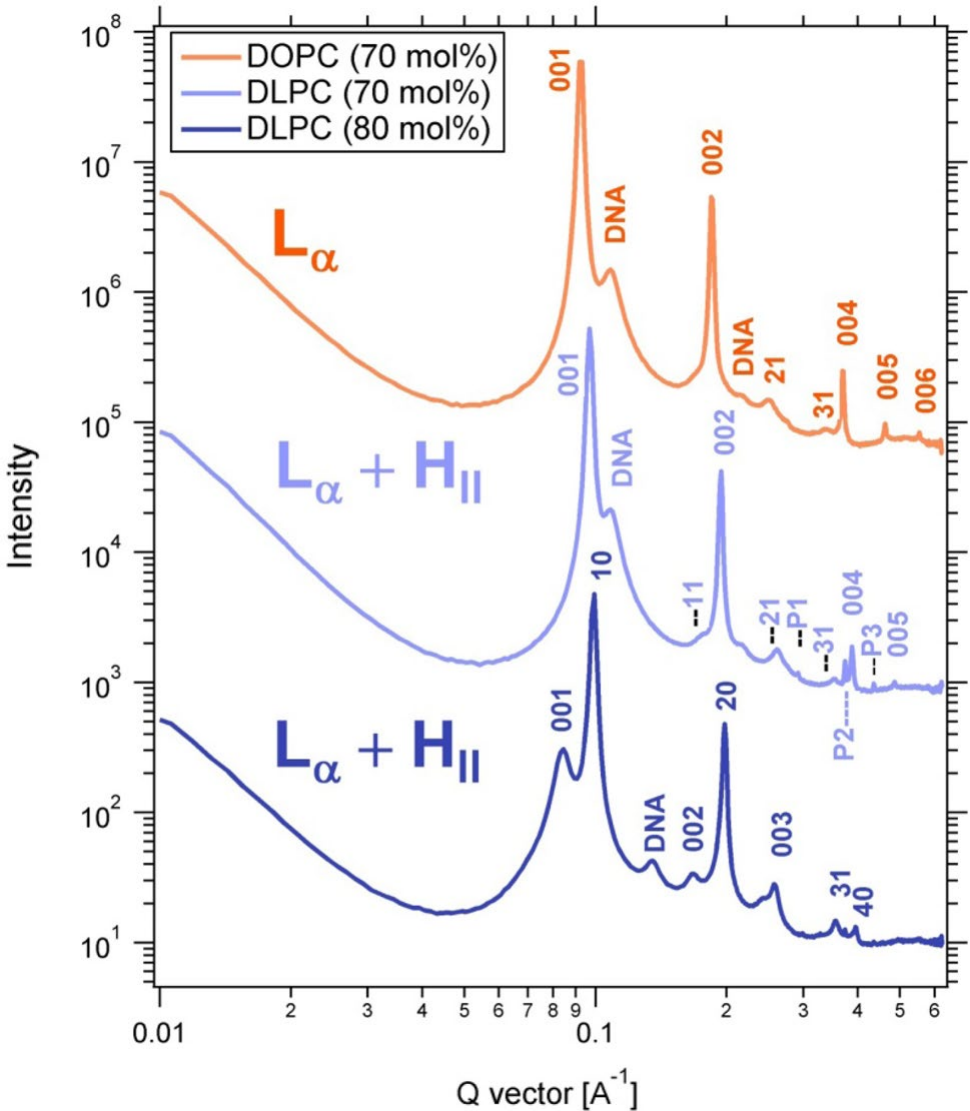
X-ray scattering profiles of CL–DNA complexes prepared from PTX-loaded CLs with high contents of DOPC or DLinPC, revealing their self-assembled structures. Peak assignments shown are for L_α_^C^ (00L), H_II_^C^ (HK), DNA–DNA interaxial spacing in the L_α_^C^ phase (DNA), and crystallized PTX (P1, P2, P3). The CLs were composed of 70 or 80 mol% neutral lipid (DOPC or DLinPC), 2 mol% PTX, and the remainder DOTAP and were complexed with calf thymus DNA at a 1:1 charge ratio.

Figure 5 shows the azimuthally-averaged scattering profiles as a function of the reciprocal lattice vector *q* for CL–DNA complexes with membrane compositions of 70 or 80 mol% neutral lipid, 2 mol% PTX, and the remainder DOTAP. Peak assignments are shown on the plots as follows: L_α_^C^ (00L: 001, 002, 003, 004, 005, 006), H_II_^C^ (HK: 10, 20, 21, 30, 31, 40), DNA–DNA interaxial spacing in the L_α_^C^ phase (DNA). Additionally, three peaks that can be assigned to crystallized PTX are visible in the sample with 70 mol% DLinPC (*q*_P1_ = 0.291 Å^−1^, *q*_P2_ = 0.374 Å^−1^, and *q*_P3_ = 0.496 Å^−1^), consistent with previous studies^48^. The L_α_^C^ phase consists of alternating lipid bilayers and DNA^103^ and the H_II_^C^ phase consists of a hexagonal arrangement of inverse cylindrical micelles with DNA inserted in their lumen^104^.

The X-ray scattering profile from CL–DNA complexes with 70 mol% DOPC shows peaks characteristic of the lamellar L_α_^C^ phase (Fig. 5, top profile). Based on this result and previous reports^108^, we expect that CL–DNA complexes with 80 mol% DOPC in the membrane would also be in the L_α_^C^ phase. In contrast, SAXS showed that CL–DNA complexes with 70 and 80 mol% of DLinPC in the membrane were in a 2-phase regime, consisting of a mixture of the lamellar (L_α_^C^) and inverted hexagonal (H_II_^C^) phases. This is expected because the cylindrical DOTAP with *C*_0_≈0 prefers the L_α_^C^ phase, whereas the inverse-cone-shaped DLinPC with *C*_0_ < 0 prefers the H_II_^C^ phase. The peak intensities (Fig. 5) reveal that the L_α_^C^ phase dominates at 70 mol% DLinPC, but at increased DLinPC content of 80 mol%, the H_II_^C^ phase is dominant.

**Figure 6:**
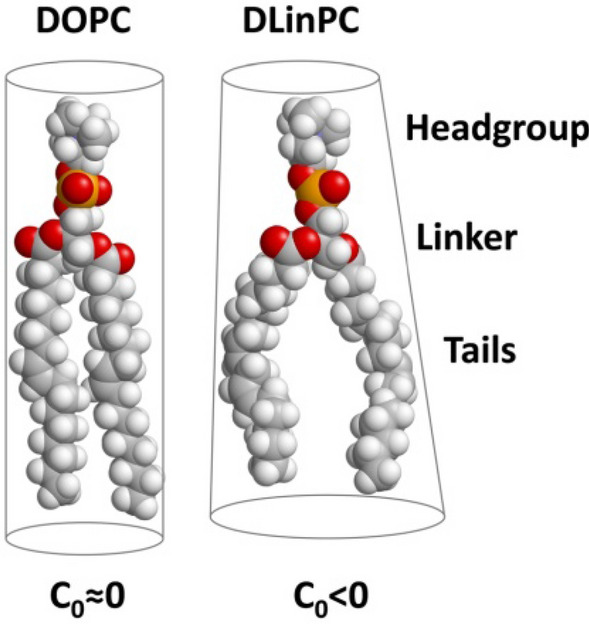
Effect of increased tail unsaturation on lipid molecular shape. The additional *cis* double bond in the linoleoyl tails induces a kink in the tails that is not readily offset by gauche conformations of single bonds in the chain. The linoleoyl tails therefore take up a greater lateral area. This changes the molecular shape, resulting in a different spontaneous curvature (*C*_0_ < 0) and thinning of the bilayer. The 3D chemical structures were rendered using RasTop 2.2 (https://www.geneinfinity.org/rastop/).

Interestingly, at 70 mol% neutral lipid (Fig. 5, top two profiles), the *q*_001_ peak of the L_α_^C^ phase in the sample containing DLinPC is observed at higher *q* (*q*_001_ = 0.0968 Å^−1^) than the peak of the sample with DOPC (*q*_001_ = 0.0931 Å^−1^). The interlayer spacing *d*_*lamellar*_ = 2π/*q*_001_ correlates to the membrane thickness. Thus, the shift in *q*_001_ indicates that the interlayer spacing drops from 67.5 to 64.9 Å when DOPC is replaced with DLinPC. DLinPC membranes are therefore thinner than DOPC membranes, which is consistent with the expectation that the added double bond forces the tails to take up more lateral width, thereby thinning the membrane (as shown in Fig. 6).

The results of the SAXS studies could indicate that the significantly improved PTX membrane solubility upon replacing oleoyl tails in CLs with linoleoyl is due to the structural transition from lipid bilayers to inverse cylindrical micelles. However, PTX solubility is actually much lower in membranes that form the H_II_^C^ instead of the L_α_^C^ phase if they consist only of oleoyl tails. This is evident from Fig. S1 in the Supplementary Information, which shows the kinetic phase diagram for PTX solubility in CLs containing 70 mol% DOPE, DOTAP and PTX. DNA complexes of these membranes form the H_II_^C^ phase (see Figure S2 in the Supplementary Information) because the headgroup of DOPE (phosphoethanolamine) is smaller than that of DOPC (phosphocholine). Therefore, improved solubility of PTX in DLinTAP/DLinPC membranes is not due to their preference for the H_II_^C^ phase but rather the different interactions of DLin tails with PTX.

### Cytotoxicity

The kinetic phase diagram data suggests that CLs composed of DLinTAP and DLinPC can stably solubilize up to nearly threefold more PTX in their membrane than those composed of DOTAP and DOPC (Fig. 4). This enhanced loading capacity will only provide significant benefits if the amount of PTX required to achieve cytotoxic efficacy against cancer cells does not change. To compare the ability of DOTAP/DOPC and DLinTAP/DLinPC PTX carriers to induce cancer cell death (i.e., their efficacy), we measured their IC50 for PTX cytotoxicity in cell viability experiments for two human cancer cell lines. The IC50 is defined as the concentration of PTX required to elicit half the maximum cytotoxic effect. These cytotoxicity experiments were conducted using sonicated CLs.

**Figure 7:**
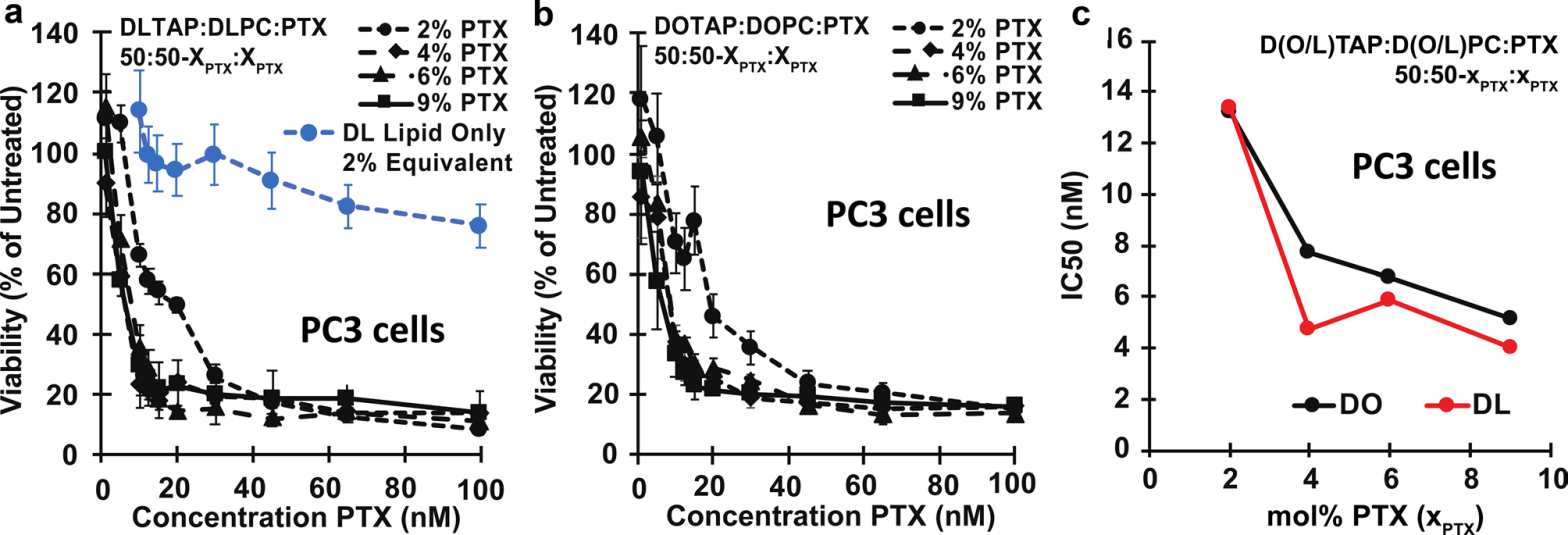
Cytotoxicity of PTX-loaded CLs against PC3 cells. (**a**) Viability of PC3 cells (relative to untreated cells) as a function of PTX concentration for cells treated with CLs of a molar composition of 50/50–x_PTX_/x_PTX_, DLinTAP/DLinPC/PTX, with x_PTX_ = 2, 4, 6, or 9 (black lines). Cell viability rapidly decreased with PTX concentration to a plateau. As a control, DLinTAP/DLinPC CLs without PTX were added to PC3 cells in amounts that match the lipid content of CLs with x_PTX_ = 2 (which have the highest lipid content) at the PTX concentrations tested (blue line). This CL-only control showed no appreciable cytotoxicity (≥ 90% viability) up to the lipid equivalent of 65 nM PTX delivered with a 2 mol% PTX formulation, demonstrating that PTX and not lipid drove cytotoxicity at the IC50 of every formulation. (**b**) Viability of PC3 cells (relative to untreated cells) as a function of PTX concentration for cells treated with control CLs of a molar composition of 50/50–x_PTX_/x_PTX_, DOTAP/DOPC/PTX, with x_PTX_ = 2, 4, 6, or 9 (black lines). Cell viability rapidly decreased with PTX concentration to a plateau. Previous work has shown that CLs alone do not contribute to cytotoxicity at the employed concentrations^48^. (**c**) Plot of IC50 values for PTX cytotoxicity against PC3 cells for the CL formulations based on lipids with linoleoyl (“DLin”, red line) and oleoyl (“DO”, black line) tails. Each IC50 was determined by fitting the corresponding cell viability curve (from parts **a** and **b**) as described in the Methods section. The IC50 decreases (efficacy increases) with increasing PTX content in both DOTAP/DOPC/PTX and DLinTAP/DLinPC/PTX formulations. The efficacy of DLinTAP/DLinPC/PTX CLs is higher than that of DOTAP/DOPC/PTX CLs at all PTX contents except 2 mol%, but difference is not significant. This shows that replacing oleoyl with linoleoyl tails maintains cytotoxic efficacy against PC3 cells while increasing PTX membrane solubility (Fig. 4).

Figure 7 shows results from cell viability experiments using CLs composed of DLinTAP/DLinPC and loaded with PTX to target prostate cancer metastasis (PC3) cells. For these measurements, we varied the PTX content of the CLs to span the range of PTX membrane solubility observed in the kinetic phase diagrams. Specifically, the PTX content ranged from 2 mol%, which is stably solubilized in both DOTAP/DOPC and DLinTAP/DLinPC CLs, up to 9 mol%, which rapidly precipitates from DOTAP/DOPC CLs and is only moderately stable in DLinTAP/DLinPC CLs. The viability of PC3 cells (normalized to untreated cells) rapidly decreases with increasing PTX concentration delivered by DLinTAP/DLinPC CLs (Fig. 7a), with most of the decrease occurring between 5 and 20 nM PTX.

To control for lipid toxicity, we also measured the cell viability for increasing concentrations of DLinTAP/DLinPC CLs without any PTX (Fig. 7a, blue curve). For comparison with PTX-loaded CLs, we incubated the cells with the amount of DLinTAP/DLinPC CLs required to deliver PTX at 2 mol% PTX content at each PTX concentration (because the formulation at 2 mol% PTX has the highest CL/PTX ratio). Lipid toxicity (> 10% drop in cell viability) was observed only at lipid concentrations equivalent to or greater than those required to deliver 65 nM PTX at 2 mol% PTX content. Because the IC50 for PTX-loaded DLinTAP/DLinPC CLs at 2 mol% PTX was 13.4 nM, more than four times lower than the PTX concentration where lipid toxicity was observed with this formulation, it is unlikely that lipid toxicity contributed to its cytotoxic efficacy. The same is true for all other formulations, considering that they delivered more PTX per lipid and had lower IC50 values than the CLs with 2 mol% PTX content.

To compare the cytotoxic efficacy of DLinTAP/DLinPC CLs to that of CL-based PTX carriers currently in clinical use, we measured the cytotoxicity of PTX-loaded DOTAP/DOPC CLs (Fig. 7b). These CLs also decreased the viability of PC3 cells (normalized to untreated cells) as a function of delivered PTX concentration, with a rapid drop of cell viability between 5 and 20 nM PTX. Steffes et al. previously demonstrated^48^ that DOTAP/DOPC CLs without PTX are not cytotoxic at any lipid concentration used in this study and therefore unlikely to affect the cytotoxicity of the PTX-loaded CLs.

To facilitate comparison of the DLinTAP/DLinPC and DOTAP/DOPC formulations, Fig. 7c plots their IC50 for cytotoxicity of PTX against PC3 cells as a function of their PTX content. The IC50 values of DLinTAP/DLinPC and DOTAP/DOPC CLs at each PTX content are very similar, demonstrating that cytotoxic efficacy is unaffected by the change in tail structure. The efficacy against PC3 cells increased (i.e., IC50 decreased) with increasing PTX content in formulations with both DO- and DLin-lipids, while previous studies had found an increase in the IC50 with PTX content for DO-lipid-based CLs^48^. A possible explanation for this is that, even at high PTX content, the PTX remained solubilized in the membranes long enough to exert its cytotoxic effects, allowing successful PTX delivery by metastable CLs. If such PTX-loaded CLs are not used immediately after preparation, however, PTX crystallizes and their efficacy drops^48^.

**Figure 8:**
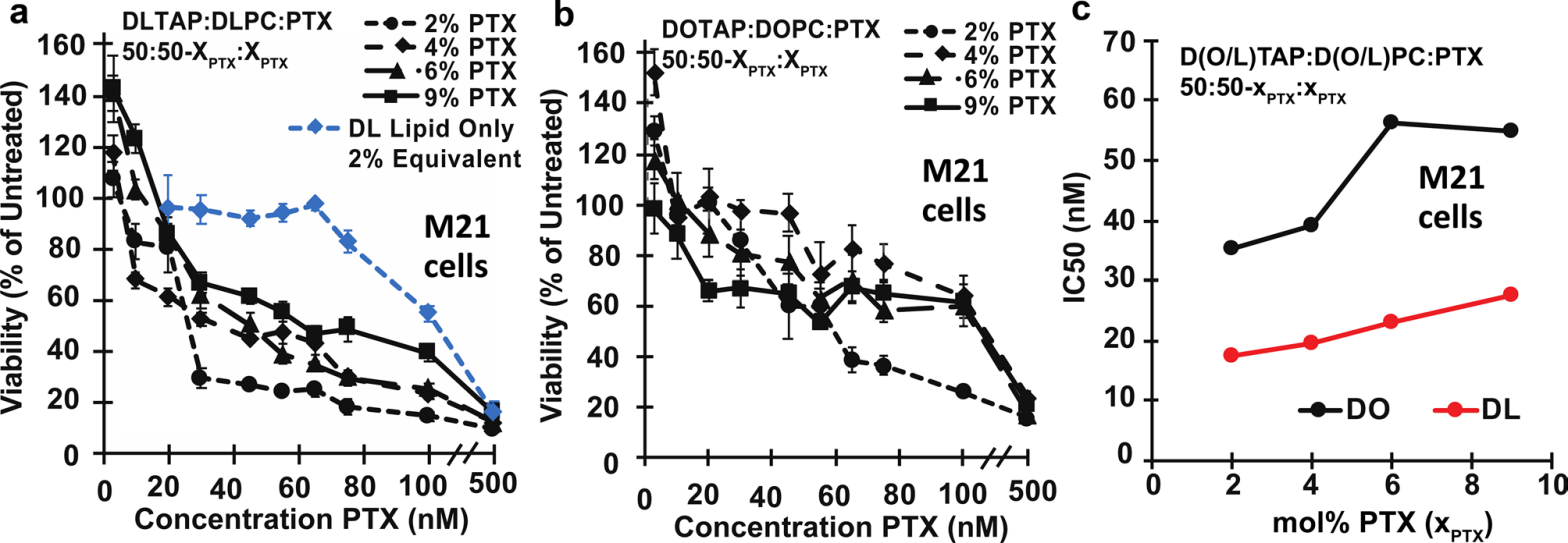
Cytotoxicity of PTX-loaded CLs against M21 cells. (**a**) Viability of M21 cells (relative to untreated cells) as a function of PTX concentration for cells treated with CLs of a molar composition of 50/50–x_PTX_/x_PTX_, DLinTAP/DLinPC/PTX, with x_PTX_ = 2, 4, 6, or 9 (black lines). Cell viability decreased with PTX concentration to a similar level as observed for PC3 cells, albeit more gradually. As a control, DLinTAP/DLinPC CLs without PTX were added to M21 cells in amounts matching the lipid content of CLs with x_PTX_ = 2 (which have the highest lipid content) at the PTX concentrations tested (blue line). The control showed no cytotoxicity (≥ 90% viability) up to the lipid equivalent of 75 nM PTX delivered with x_PTX_ = 2. This suggests that PTX, not lipid, drove cytotoxicity at the IC50 of every formulation because CL cytotoxicity manifests only at concentrations at least four times the IC50 (see part **c**). (**b**) Viability of M21 cells (relative to untreated cells) as a function of PTX concentration for cells treated with control CLs of a molar composition of 50/50–x_PTX_/x_PTX_, DOTAP/DOPC/PTX, with x_PTX_ = 2, 4, 6, or 9 (black lines). The decrease in cell viability with PTX concentration is less steep than in part A, suggesting a lower efficacy. At the employed concentrations, CLs without PTX do not elicit cytotoxicity^48^. (**c**) Plot of IC50 values for PTX cytotoxicity against M21 cells for the CL formulations based on lipids with linoleoyl (“DLin”, red line) and oleoyl (“DO”, black line) tails. Each IC50 was determined by fitting the corresponding cell viability curve (from parts **a** and **b**) as described in the Methods section. Importantly, the efficacy of the DLin formulations was about two-fold higher (their IC50 values were two-fold lower) than that of the corresponding DO formulations. This effect amplifies the benefits of increased PTX membrane solubility (Fig. 4). In contrast to PC3 cells, the IC50 increases (efficacy decreases) with increasing PTX content for both DO and DLin formulations. This effect is less pronounced for the DLin formulations, and the IC50 of the DLin formulation at x_PTX_ = 9 is lower than that of the DO formulation at x_PTX_ = 2.

We also investigated the efficacy of PTX-loaded DLinTAP/DLinPC CLs against a metastatic melanoma (M21) cell line, using the same range of CL formulations and PTX contents as for the PC3 cell line (Fig. 8a). Compared to PC3 cells, the viability of M21 cells decreased more gradually, with most of the drop in viability occurring between 10 and 65 nM PTX, depending on the specific formulation. This is consistent with prior investigations using PTX-loaded DOTAP/DOPC CLs^48^.

We again assessed DLinTAP/DLinPC lipid toxicity using a CL-only control. DLinTAP/DLinPC CLs without PTX caused > 10% drop in cell viability only at or above lipid concentrations equivalent to those present when 75 nM PTX is delivered by CLs loaded with 2 mol% PTX (Fig. 8a, blue line). In contrast, the IC50 for the same CLs with PTX is 17.6 nM PTX, about four times lower. It is therefore unlikely that lipid toxicity contributed significantly to the measured IC50 values for PTX-loaded CLs at any PTX content, given that the lipid/PTX ratio for the formulations decreased more than the IC50 increased (see below).

Viability of M21 cells as a function of delivered PTX concentration for the positive control, DOTAP/DOPC CLs loaded with 2 to 9 mol% PTX, is shown in Fig. 8b. The decrease in cell viability with PTX concentration is very gradual and slower than that for PTX-loaded DLinTAP/DLinPC CLs (Fig. 8a), suggesting that DLinTAP/DLinPC CLs induced cytotoxicity more effectively than DOTAP/DOPC CLs when used against M21 cells. According to literature data, DOTAP/DOPC CLs without PTX are not cytotoxic at concentrations well above those used in this experiment^48^.

For a more quantitative comparison, Fig. 8c plots the IC50 for PTX cytotoxicity against M21 cells of the investigated formulations as a function of their membrane PTX content. The IC50 of PTX-loaded DLinTAP/DLinPC CLs was at least twofold lower than that of DOTAP/DOPC CLs at every PTX content tested. Because toxicity of PTX-free DLinTAP/DLinPC CLs is only observed well above the IC50 of those formulations, this improved efficacy is unlikely to be due to differences in lipid toxicity. Interestingly, the efficacy of formulations against M21 cells increases (their IC50 decreases) with PTX membrane solubility. This is in line with expectations based on previous findings regarding the correlation of PTX solubility to cytotoxic efficacy^48^.

Taken together, PTX-loaded DLinTAP/DLinPC CLs show the same or better efficacy against cancer cells as the established DOTAP/DOPC/PTX formulation (Endo-TAG1), in addition to the improved drug loading capacity demonstrated by the kinetic phase diagrams.

## Conclusion

Our results have demonstrated how modifying lipid tail structure can improve cationic liposome (CL)-based carriers of cancer chemotherapy drug paclitaxel (PTX), by revealing two significant advantages over CL formulations that mimic the Endo-TAG1 (DOTAP/DOPC/PTX) vectors currently in phase III trials. The data show that replacement of lipid tails bearing one *cis* double bond (oleoyl tails, DO lipids) with tails bearing two *cis* double bonds (linoleoyl tails, DLin lipids) enhances solubility of PTX in CL vectors and impacts efficacy against in two human cancer cell lines. The increased tail unsaturation significantly improved PTX membrane solubility in the CLs from ≈3 mol% to ≈8 mol%. This improves the drug loading capacity of the CL vector, allowing the same amount of PTX to be delivered with much less lipid. Minimizing the amount of cationic lipids in in vivo delivery applications is highly desirable not only because it reduces cost but also because such cationic lipids can evoke a cellular immune response with measurable amounts of secreted interleukins^109^.

In addition to the important benefits derived from the reduced amount of cationic lipid needed to deliver PTX, a further significant finding of our study is that CL vectors based on DLin lipids have either similar or even improved efficacy when compared to vectors based on DO lipids, as evident by their IC50 of PTX cytotoxicity against prostate cancer (PC3) and melanoma (M21) cell lines, respectively. An increased efficacy allows reducing the amount of both lipid and PTX (or, alternatively, an enhanced effectiveness against cancer cells if the dose of PTX is kept constant).

Our results pave the path for in vivo studies to determine whether improved CL carrier properties, for PTX delivery to human cancer cells in vitro, translate to substantially improved outcomes in vivo. In particular, it will be important to establish whether the 8 to 10 h solubility window, found for CLs with significantly larger PTX content, is sufficient for drug delivery in vivo.

We expect that this work will motivate future studies using chemical modifications of lipid structure as well as computational modeling to further explore how altering lipid tails affects their molecular affinity to PTX. This, when combined with kinetic phase diagrams as presented here, should lead to a comprehensive understanding of how lipid shape and tail structure correlates to PTX membrane solubility, paving the way to improved cancer therapeutics.

## Supplementary Information

Supplementary Information 1.

## Data Availability

The datasets generated during and/or analyzed during the current study are available from the corresponding author on reasonable request.

## References

[1] Wani MC, Taylor HL, Wall ME, Coggon P, McPhail AT (1971). Plant antitumor agents. VI. Isolation and structure of taxol, a novel antileukemic and antitumor agent from Taxus brevifolia. J. Am. Chem. Soc..

[2] Jordan MA, Wilson L (2004). Microtubules as a target for anticancer drugs. Nat. Rev. Cancer.

[3] Weaver BA (2014). How Taxol/paclitaxel kills cancer cells. Mol. Biol. Cell.

[4] Rowinsky EK, Donehower RC (1995). Paclitaxel (Taxol). N. Engl. J. Med..

[5] Markman M, Mekhail TM (2002). Paclitaxel in cancer therapy. Expert Opin. Pharmacother..

[6] Ramalingam S, Belani CP (2004). Paclitaxel for non-small cell lung cancer. Expert Opin. Pharmacother..

[7] Hironaka S (2006). Weekly paclitaxel as second-line chemotherapy for advanced or recurrent gastric cancer. Gastric Cancer.

[8] Sakamoto J, Matsui T, Kodera Y (2009). Paclitaxel chemotherapy for the treatment of gastric cancer. Gastric Cancer.

[9] Moxley KM, McMeekin DS (2010). Endometrial carcinoma: a review of chemotherapy, drug resistance, and the search for new agents. Oncologist.

[10] World Health Organization. *WHO Model Lists of Essential Medicines*. http://www.who.int/medicines/publications/essentialmedicines/en/.

[11] Yvon A-MC, Wadsworth P, Jordan MA (1999). Taxol suppresses dynamics of individual microtubules in living human tumor cells. Mol. Biol. Cell.

[12] Schiff PB, Fant J, Horwitz SB (1979). Promotion of microtubule assembly in vitro by taxol. Nature.

[13] Jordan MA (1996). Mitotic block induced in HeLa cells by low concentrations of paclitaxel (Taxol) results in abnormal mitotic exit and apoptotic cell death. Cancer Res..

[14] Jordan MA, Toso RJ, Thrower D, Wilson L (1993). Mechanism of mitotic block and inhibition of cell proliferation by taxol at low concentrations. Proc. Natl. Acad. Sci. U. S. A..

[15] Torchilin VP (2001). Structure and design of polymeric surfactant-based drug delivery systems. J. Control. Release.

[16] Drummond DC, Meyer O, Hong K, Kirpotin DB, Papahadjopoulos D (1999). Optimizing liposomes for delivery of chemotherapeutic agents to solid tumors. Pharmacol. Rev..

[17] Bristol-Myers Squibb Company. Taxol [package insert]. Princeton, NJ: April 2011. http://www.accessdata.fda.gov/drugsatfda_docs/label/2011/020262s049lbl.pdf.

[18] Dorr RT (1994). Pharmacology and toxicology of cremophor EL diluent. Ann. Pharmacother..

[19] Weiss RB (1990). Hypersensitivity reactions from taxol. J. Clin. Oncol..

[20] Gelderblom H, Verweij J, Nooter K, Sparreboom A (2001). Cremophor EL: the drawbacks and advantages of vehicle selection for drug formulation. Eur. J. Cancer.

[21] Sofias AM, Dunne M, Storm G, Allen C (2017). The battle of “nano” paclitaxel. Adv. Drug Deliv. Rev..

[22] Surapaneni MS, Das SK, Das NG (2012). Designing paclitaxel drug delivery systems aimed at improved patient outcomes: current status and challenges. ISRN Pharmacol..

[23] Tibbitt MW, Dahlman JE, Langer R (2016). Emerging frontiers in drug delivery. J. Am. Chem. Soc..

[24] Ma P, Mumper RJ (2013). Paclitaxel nano-delivery systems: a comprehensive review. J. Nanomed. Nanotechnol..

[25] Rosenblum D, Joshi N, Tao W, Karp JM, Peer D (2018). Progress and challenges towards targeted delivery of cancer therapeutics. Nat. Commun..

[26] Bernabeu E, Cagel M, Lagomarsino E, Moretton M, Chiappetta DA (2017). Paclitaxel: what has been done and the challenges remain ahead. Int. J. Pharm..

[27] Wang F, Porter M, Konstantopoulos A, Zhang P, Cui H (2017). Preclinical development of drug delivery systems for paclitaxel-based cancer chemotherapy. J. Control. Release.

[28] Dranitsaris G (2016). Abraxane versus Taxol for patients with advanced breast cancer: a prospective time and motion analysis from a Chinese health care perspective. J. Oncol. Pharm. Pract..

[29] Rugo HS (2015). Randomized phase III trial of paclitaxel once per week compared with nanoparticle albumin-bound nab-paclitaxel once per week or ixabepilone with bevacizumab as first-line chemotherapy for locally recurrent or metastatic breast cancer: CALGB 40502/NCCTG N063H (alliance). J. Clin. Oncol..

[30] Gradishar WJ (2005). Phase III trial of nanoparticle albumin-bound paclitaxel compared with polyethylated castor oil-based paclitaxel in women with breast cancer. J. Clin. Oncol..

[31] Hersh EM (2015). A randomized, controlled phase III trial of nab-Paclitaxel versus dacarbazine in chemotherapy-naïve patients with metastatic melanoma. Ann. Oncol..

[32] Allen TM, Cullis PR (2013). Liposomal drug delivery systems: from concept to clinical applications. Adv. Drug Deliv. Rev..

[33] Sercombe L (2015). Advances and challenges of liposome assisted drug delivery. Front. Pharmacol..

[34] Safinya CR, Ewert KK, Majzoub RN, Leal C (2014). Cationic liposome-nucleic acid complexes for gene delivery and gene silencing. New J. Chem..

[35] Torchilin VP (2005). Recent advances with liposomes as pharmaceutical carriers. Nat. Rev. Drug Discov..

[36] Teo PY, Cheng W, Hedrick JL, Yang YY (2016). Co-delivery of drugs and plasmid DNA for cancer therapy. Adv. Drug Deliv. Rev..

[37] Koudelka Š, Turánek J (2012). Liposomal paclitaxel formulations. J. Control. Release.

[38] Fasol U (2012). Vascular and pharmacokinetic effects of EndoTAG-1 in patients with advanced cancer and liver metastasis. Ann. Oncol..

[39] Campbell RB, Ying B, Kuesters GM, Hemphill R (2009). Fighting cancer: from the bench to bedside using second generation cationic liposomal therapeutics. J. Pharm. Sci..

[40] Strieth S (2004). Neovascular targeting chemotherapy: Encapsulation of paclitaxel in cationic liposomes impairs functional tumor microvasculature. Int. J. Cancer.

[41] Strieth S (2008). Tumor-selective vessel occlusions by platelets after vascular targeting chemotherapy using paclitaxel encapsulated in cationic liposomes. Int. J. Cancer.

[42] Kunstfeld R (2003). Paclitaxel encapsulated in cationic liposomes diminishes tumor angiogenesis and melanoma growth in a “humanized” SCID mouse model. J. Invest. Dermatol..

[43] Schmitt-Sody M (2003). Neovascular targeting therapy: paclitaxel encapsulated in cationic liposomes improves antitumoral efficacy. Clin. Cancer Res..

[44] Feng L, Mumper RJ (2013). A critical review of lipid-based nanoparticles for taxane delivery. Cancer Lett..

[45] Huang S-T (2018). Liposomal paclitaxel induces fewer hematopoietic and cardiovascular complications than bioequivalent doses of Taxol. Int. J. Oncol..

[46] Lim SB, Banerjee A, Önyüksel H (2012). Improvement of drug safety by the use of lipid-based nanocarriers. J. Control. Release.

[47] Fritze A, Hens F, Kimpfler A, Schubert R, Peschka-Süss R (2006). Remote loading of doxorubicin into liposomes driven by a transmembrane phosphate gradient. Biochim. Biophys. Acta, Biomembr..

[48] Steffes VM (2017). Distinct solubility and cytotoxicity regimes of paclitaxel-loaded cationic liposomes at low and high drug content revealed by kinetic phase behavior and cancer cell viability studies. Biomaterials.

[49] Campbell RB, Balasubramanian SV, Straubinger RM (2001). Influence of cationic lipids on the stability and membrane properties of paclitaxel-containing liposomes. J. Pharm. Sci..

[50] Bernsdorff C, Reszka R, Winter R (1999). Interaction of the anticancer agent taxol (paclitaxel) with phospholipid bilayers. J. Biomed. Mater. Res..

[51] Campbell RB (2002). Cationic charge determines the distribution of liposomes between the vascular and extravascular compartments of tumors. Cancer Res..

[52] Eichhorn ME (2010). Vascular targeting by EndoTAG-1 enhances therapeutic efficacy of conventional chemotherapy in lung and pancreatic cancer. Int. J. Cancer.

[53] Thurston G (1998). Cationic liposomes target angiogenic endothelial cells in tumors and chronic inflammation in mice. J. Clin. Invest..

[54] Ho EA (2010). Characterization of cationic liposome formulations designed to exhibit extended plasma residence times and tumor vasculature targeting properties. J. Pharm. Sci..

[55] Bode C (2009). Paclitaxel encapsulated in cationic liposomes: a new option for neovascular targeting for the treatment of prostate cancer. Oncol. Rep..

[56] Dellian M, Yuan F, Trubetskoy VS, Torchilin VP, Jain RK (2000). Vascular permeability in a human tumour xenograft: molecular charge dependence. Br. J. Cancer.

[57] Foldvari M (2016). Non-viral gene therapy: gains and challenges of non-invasive administration methods. J. Control. Release.

[58] Geinguenaud F, Guenin E, Lalatonne Y, Motte L (2016). Vectorization of nucleic acids for therapeutic approach: tutorial review. ACS Chem. Biol..

[59] Young SWS, Stenzel M, Jia-Lin Y (2016). Nanoparticle-siRNA: a potential cancer therapy?. Crit. Rev. Oncol..

[60] Ozcan G, Ozpolat B, Coleman RL, Sood AK, Lopez-Berestein G (2015). Preclinical and clinical development of siRNA-based therapeutics. Adv. Drug Deliv. Rev..

[61] Yin H, Kauffman KJ, Anderson DG (2017). Delivery technologies for genome editing. Nat. Rev. Drug Discov..

[62] Rupaimoole R, Slack FJ (2017). MicroRNA therapeutics: towards a new era for the management of cancer and other diseases. Nat. Rev. Drug Discov..

[63] Guan S, Rosenecker J (2017). Nanotechnologies in delivery of mRNA therapeutics using nonviral vector-based delivery systems. Gene Ther..

[64] Wan C, Allen TM, Cullis PR (2014). Lipid nanoparticle delivery systems for siRNA-based therapeutics. Drug Deliv. Transl. Res..

[65] Gindy ME, Leone AM, Cunningham JJ (2012). Challenges in the pharmaceutical development of lipid-based short interfering ribonucleic acid therapeutics. Expert Opin. Drug Deliv..

[66] Yin H (2014). Non-viral vectors for gene-based therapy. Nat. Rev. Genet..

[67] Wang Y, Miao L, Satterlee A, Huang L (2015). Delivery of oligonucleotides with lipid nanoparticles. Adv. Drug Deliv. Rev..

[68] Ewert KK (2016). Synthesis of linear and cyclic peptide–PEG–lipids for stabilization and targeting of cationic liposome–DNA complexes. Bioorg. Med. Chem. Lett..

[69] Majzoub RN, Ewert KK, Safinya CR (2016). Cationic liposome–nucleic acid nanoparticle assemblies with applications in gene delivery and gene silencing. Philos. Trans. R. Soc., A..

[70] Gindy ME (2014). Mechanism of macromolecular structure evolution in self-assembled lipid nanoparticles for siRNA delivery. Langmuir.

[71] Guo X, Huang L (2012). Recent advances in nonviral vectors for gene delivery. Acc. Chem. Res..

[72] Safinya CR, Ewert KK (2012). Materials chemistry: liposomes derived from molecular vases. Nature.

[73] Pecot CV, Calin GA, Coleman RL, Lopez-Berestein G, Sood AK (2011). RNA interference in the clinic: challenges and future directions. Nat. Rev. Cancer.

[74] Lares MR, Rossi JJ, Ouellet DL (2010). RNAi and small interfering RNAs in human disease therapeutic applications. Trends Biotechnol..

[75] Li S-D, Huang L (2007). Non-viral is superior to viral gene delivery. J. Control. Release.

[76] Leal C, Ewert KK, Shirazi RS, Bouxsein NF, Safinya CR (2011). Nanogyroids incorporating multivalent lipids: enhanced membrane charge density and pore forming ability for gene silencing. Langmuir.

[77] Leal C, Bouxsein NF, Ewert KK, Safinya CR (2010). Highly efficient gene silencing activity of siRNA embedded in a nanostructured gyroid cubic lipid matrix. J. Am. Chem. Soc..

[78] Bielke, W. & Erbacher, C. in *Top. Curr. Chem*., Vol. 296 (Springer, 2010). 21504097

[79] Ewert KK (2010). Cationic liposome-nucleic acid complexes for gene delivery and silencing: pathways and mechanisms for plasmid DNA and siRNA. Top. Curr. Chem..

[80] Huang L, Hung MC, Wagner E (2005). Advances in Genetics.

[81] Ewert KK, Ahmad A, Evans HM, Safinya CR (2005). Cationic lipid-DNA complexes for non-viral gene therapy: relating supramolecular structures to cellular pathways. Expert Opin. Biol. Ther..

[82] Fetterly GJ, Straubinger RM (2003). Pharmacokinetics of paclitaxel-containing liposomes in rats. AAPS PharmSci.

[83] Sharma A, Sharma US, Straubinger RM (1996). Paclitaxel-liposomes for intracavitary therapy of intraperitoneal P388 leukemia. Cancer Lett..

[84] Sharma A, Straubinger RM (1994). Novel taxol formulations: preparation and characterization of taxol-containing liposomes. Pharm. Res..

[85] Castro, J. S., Tapia, L. V., Silveyra, R. A., Martinez, C. A. & Deymier, P. A. in *Current Cancer Treatment—Novel Beyond Conventional Approaches* (ed. Özdemir, Ö.) 767–782 (InTech Open, 2011).

[86] Que C, Gao Y, Raina SA, Zhang GGZ, Taylor LS (2018). Paclitaxel crystal seeds with different intrinsic properties and their impact on dissolution of paclitaxel-HPMCAS amorphous solid dispersions. Cryst. Growth Des..

[87] Hong S-S (2016). Development of paclitaxel-loaded liposomal nanocarrier stabilized by triglyceride incorporation. Int. J. Nanomed..

[88] Kannan V, Balabathula P, Divi MK, Thoma LA, Wood GC (2015). Optimization of drug loading to improve physical stability of paclitaxel-loaded long-circulating liposomes. J. Liposome Res..

[89] Kan P, Tsao C-W, Wang A-J, Su W-C, Liang H-F (2011). A liposomal formulation able to incorporate a high content of paclitaxel and exert promising anticancer effect. J. Drug Deliv..

[90] Yang T (2007). Enhanced solubility and stability of PEGylated liposomal paclitaxel: in vitro and in vivo evaluation. Int. J. Pharm..

[91] Yang T (2007). Preparation and evaluation of paclitaxel-loaded PEGylated immunoliposome. J. Control. Release.

[92] Yang T (2007). Antitumor effect of paclitaxel-loaded PEGylated immunoliposomes against human breast cancer cells. Pharm. Res..

[93] Zhang JA (2005). Development and characterization of a novel Cremophor EL free liposome-based paclitaxel (LEP-ETU) formulation. Eur. J. Pharm. Biopharm..

[94] Awada A (2014). A randomized controlled phase II trial of a novel composition of paclitaxel embedded into neutral and cationic lipids targeting tumor endothelial cells in advanced triple-negative breast cancer (TNBC). Ann. Oncol..

[95] Balasubramanian SV, Straubinger RM (1994). Taxol-lipid interactions: taxol-dependent effects on the physical properties of model membranes. Biochemistry.

[96] Koudelka Š (2010). Liposomes with high encapsulation capacity for paclitaxel: preparation, characterisation and *in vivo* anticancer effect. J. Pharm. Sci..

[97] Yingchoncharoen P, Kalinowski DS, Richardson DR (2016). Lipid-based drug delivery systems in cancer therapy: what is available and what is yet to come. Pharmacol. Rev..

[98] Hoy SM (2018). Patisiran: first global approval. Drugs.

[99] Begin ME, Ells G, Horrobin DF (1988). Polyunsaturated fatty acid-induced cytotoxicity against tumor cells and its relationship to lipid peroxidation. JNCI, J. Natl. Cancer Inst..

[100] Alaarg A (2016). Docosahexaenoic acid liposomes for targeting chronic inflammatory diseases and cancer: an in vitro assessment. Int. J. Nanomed..

[101] Steffes, V. M. Designing Lipid Nanoparticles Toward Targeted Drug Delivery: Fundamental Studies Identify Key Compositional Properties to Improve Formulations for the Hydrophobic Cancer Drug Paclitaxel. PhD thesis, University of California (2019).

[102] Leventis R, Silvius JR (1990). Interactions of mammalian cells with lipid dispersions containing novel metabolizable cationic amphiphiles. Biochim. Biophys. Acta, Biomembr..

[103] Rädler JO, Koltover I, Salditt T, Safinya CR (1997). Structure of DNA-cationic liposome complexes: DNA intercalation in multilamellar membranes in distinct interhelical packing regimes. Science.

[104] Koltover I, Salditt T, Rädler JO, Safinya CR (1998). An inverted hexagonal phase of cationic liposome-DNA complexes related to DNA release and delivery. Science.

[105] Ewert KK (2006). A columnar phase of dendritic lipid-based cationic liposome-DNA complexes for gene delivery: hexagonally ordered cylindrical micelles embedded in a DNA honeycomb lattice. J. Am. Chem. Soc..

[106] Israelachvili JN (2011). Intermolecular and Surface Forces.

[107] Wadsäter M, Barauskas J, Nylander T, Tiberg F (2014). Formation of highly structured cubic micellar lipid nanoparticles of soy phosphatidylcholine and glycerol dioleate and their degradation by triacylglycerol lipase. ACS Appl. Mater. Interfaces.

[108] Salditt T, Koltover I, Rädler JO, Safinya CR (1997). Two-dimensional smectic ordering of linear DNA chains in self-assembled DNA-cationic liposome mixtures. Phys. Rev. Lett..

[109] Kedmi R, Ben-Arie N, Peer D (2010). The systemic toxicity of positively charged lipid nanoparticles and the role of Toll-like receptor 4 in immune activation. Biomaterials.

